# Retrospective study of Dana Farber Consortium Protocol in newly diagnosed Egyptian adolescents and young adults with acute lymphoblastic leukemia: Tanta experience

**DOI:** 10.1186/s43046-021-00064-6

**Published:** 2021-04-07

**Authors:** Hossam Eldin A. Elashtokhy, Heba E. Elgohary, Basant B. Eldeep, Sally M. Gaber, Tamer A. Elbedewy

**Affiliations:** 1 Medical Oncology Department, Tanta Cancer Center, Tanta, Egypt; 2 Internal Medicine Department, Faculty of Medicine, Tanta University, Tanta, Egypt

**Keywords:** Acute lymphoblastic leukemia (ALL), Dana Farber Consortium Protocol (DFCP), Adolescents and young adults (AYA), Event-free survival (EFS), Overall survival (OS)

## Abstract

**Background:**

Intensive acute lymphoblastic leukemia (ALL) regimens in children improve the 5-year event-free survival (EFS) to reach ~ 90%. Adolescents and young adults (AYA) have EFS (30% to 45%). Young AYA ALL patients treated with pediatric chemotherapy protocols such as Dana Farber Consortium Protocol (DFCP) experience a better prognosis. This study aimed to assess the efficacy [EFS and overall survival (OS)] and the toxicity of DFCP in the treatment of Egyptian AYA with newly diagnosed ALL. A retrospective study was performed on 41 patients with newly diagnosed ALL (15 and 39 years) who were treated with DFCP. EFS and OS were estimated using the Kaplan-Meier method.

**Results:**

Thirty-eight patients (92.68%) achieved complete remission (CR). Eleven patients (26.83%) relapsed. Ten (24.39%) patients died. One, two, and three years of EFS were 75.61%, 72.91%, and 67.51% respectively. One, two, and three years OS were 85.3%, 77.26%, and 74.39% respectively. Neutropenia was the most common adverse event observed in 100% of patients.

**Conclusion:**

DFCP can be considered as an effective ALL protocol for the AYA group of patients with good CR, EFS, and OS rates. DFCP seemed to be feasible in AYA despite the toxicities experienced.

## Background

Acute lymphoblastic leukemia (ALL) is a heterogeneous lymphoid neoplasm characterized by a malignant transformation and proliferation of lymphoid precursors in the bone marrow, blood, and many other organs [1]. ALL is the most common pediatric malignancy represents about 75% of acute leukemia among this age group; while in adults; ALL represents about 20% of all leukemia [2].

The survival rates for ALL patients have improved significantly due to advanced diagnostics especially for minimal residual disease, new targeted therapy, and the use of allogeneic bone marrow transplantation (BMT) [3].

In general, the estimated 5-year survival for ALL is 68.6% [4]. Intensive ALL chemotherapy regimens in children improve the 5-year event-free survival (EFS) to reach ~ 90% [5]. Adolescents and young adults (AYA) (15–39 years) historically have much poorer outcomes, with EFS (30% to 45%) due to possible disease and patient biology differences [6, 7]. The identification of special characters of AYA with ALL has led to an improvement of EFS, with EFS now ~ 70% [8]. Young AYA (15–21 years at diagnosis) ALL patients treated with pediatric chemotherapy protocols such as Dana Farber Consortium Protocol (DFCP) experience better prognosis when compared with AYA treated with adult protocols [9].

So, this study aimed to assess the efficacy [Event free survival (EFS) and overall survival (OS)] and the toxicity of the DFCP in the treatment of Egyptian adolescents and young adults with newly diagnosed acute lymphoblastic leukemia.

## Methods

### Patients

This was a retrospective study performed on 41 patients with newly diagnosed ALL who were treated with DFCP selected consecutively and diagnosed between January 2014 and December 2017 at Tanta Cancer Center (acute leukemia unit) and Hematology Unit, Internal Medicine Department, Faculty of Medicine, Tanta University.

The diagnosis of ALL was based on the characteristic presence of 20% or more lymphoblast in bone marrow (BM) examination and was confirmed by immunophenotyping and cytogenetic and /or molecular studies [10].

### Eligibility criteria

The patients included in this study were newly diagnosed ALL with no prior chemotherapy, aged between 15 and 39 years.

ALL patients with performance status Eastern Cooperative Oncology Group (ECOG) (3 or more) and patients with chronic renal, hepatic, or uncontrolled severe cardiovascular disease and pregnant women were excluded. Patients with an uncontrolled active infection, human immunodeficiency virus, active psychiatric illness, cerebrovascular accident or hemorrhage, or prior history of pancreatitis were also excluded.

### Treatment protocol: (Table 1)

All patients received DFCP with supportive therapy [11]. Imatinib or dasatinib was added to the protocol in the case of Philadelphia positive [12].

**Table 1:** Dana Farber Consortium Protocol used in the study [11, 12]

Phase	Duration	Drug	Dose	Route of administration	Days	Notes
Phase I induction	4 weeks	Prednisone	40 mg/m^2^	Oral	D1-29	
		Vincristine	1.4 mg/m^2^	Intravenous	D1,8,15,22	Maximum dose 2 mg
		Doxorubicin	30 mg/m^2^	Intravenous	D1,2	
		Methotrexate	1.5 g/m^2^	Intravenous	D3	leucovorin rescue
		L-asparaginase	25,000 IU/m^2^	Intramuscular	D5	Ph-ve patients only
		Cytarabine	40 mg	Intrathecal	D1	
		Methotrexate Cytarabine Hydrocortisone	12 mg 40 mg 15 mg	Intrathecal	D15,29	
		Imatinib	600 mg		D3-15	Ph +ve patients only
Phase II induction and CNS therapy	3 weeks	Vincristine	1.4 mg/m^2^	Intravenous	D1	Maximum dose 2 mg
		Doxorubicin	30 mg/m^2^	Intravenous	D1	
		6-Mercaptopurine	50 mg/m^2^	Oral	D1-14	
		Methotrexate Cytarabine Hydrocortisone	12 mg 40 mg 15 mg	Intrathecal	D1, 4, 8, 11	
		Cranial irradiation	1200 centigray		Over 8 days	With very high initial total leucocytic count or CNS infiltration unless patient will have BMT
Phase III Intensification therapy	10 cycles (cycle every 3 weeks)	Dexamethasone	9 mg/m^2^ twice daily	Oral	D1-5	
		6-Mercaptopurine	50 mg/m^2^	Oral	D1-14	
		Vincristine	1.4 mg/m^2^	Intravenous	D1	Maximum dose 2 mg
		l-asparaginase	12,500 IU/m^2^	Intramuscular	D1, 8, 15	
		Doxorubicin	30 mg/m^2^	Intravenous	D1	In the first 7 cycles
		Methotrexate	30 mg/m^2^	Intramuscular or oral	D2, 9, 16	In last 3 cycles
		Methotrexate Cytarabine Hydrocortisone	12 mg 40 mg 15 mg	Intrathecal	D1	6^th^ cycle only
Phase IV Continuation therapy	24 cycles (cycle every 3 weeks)	Dexamethasone	6 mg/m^2^ twice daily	Oral	D1-5	
		6-Mercaptopurine	50 mg/m^2^	Oral	D1-14	
		Vincristine	1.4 mg/m^2^	Intravenous	D1	Maximum dose 2 mg
		Methotrexate	30 mg/m^2^	Intramuscular or oral	Weekly	
		Methotrexate Cytarabine Hydrocortisone	12 mg 40 mg 15 mg	Intrathecal	Every 18 weeks at the start of the cycle	

*BMT* bone marrow transplantation, *CNS* central nervous system, *D* day, *Ph* Philadelphia

### Data collection

Data were collected by reviewing patients’ records. Records with incomplete data (four patients) were omitted from the study. Every patient has a file with a private code number to ensure the privacy of our patients. All patients’ data were handled according to ethical standards in accordance with the Declaration of Helsinki. Signed informed consents from all alive and still followed up included patients were taken before the starting of data collection.

Data that were collected included age, sex, history, physical examination, and initial laboratory investigations including complete blood count (CBC), BM aspiration, immunophenotyping, cytogenetics study, and BCR-ABL1 test.

### Follow-up

While patients on therapy complete blood counts and BM aspirations with other routine investigations were done for assessing the patient’s response. The patients were followed up weekly by complete blood counts for 33 weeks during phase II induction, central nervous system (CNS) prophylaxis, and phase III intensifications therapy. After the completion, patients were followed up by the complete physical examination, blood cell counts, and routine chemistry; every month during the first year. Thereafter, every 3 months in the 2^nd^ year then half-yearly from the 3^rd^ year. BM aspiration was done every 6 months or as needed clinical for 5 years. BCR-ABL1 was done periodically every 3 months for Philadelphia positive patients [8].

Minimal residual disease (MRD) was evaluated post-induction and those who achieved more than 0.1% were referred for allogeneic BMT with Philadelphia positive and relapsed patients [8].

### Response to treatment [8]

#### Complete remission

Less than 5% blasts in BM, no peripheral blood blasts, absolute neutrophil count at least 1 × 10^9^/L, platelet counts of at least 100 × 10^9^/L, and no extra-medullary disease, if only platelets count and/or absolute neutrophil count not reach to the target complete remission (CR) with incomplete blood count recovery.

#### Refractory disease

Failure to achieve CR at the end of induction.

#### Relapsed disease

The reappearance of blasts in the blood or bone marrow (> 5%) or any extra-medullary site after CR.

### Outcomes

Event-free survival (EFS) was calculated from the date of ALL diagnosis till the date of treatment failure, ALL relapse, last follow-up, or death. Overall survival (OS) was calculated from the date of ALL diagnosis to the date of last follow-up or death.

### Statistical analysis

The collected data were analyzed using SPSS version 23 software (SPSS Inc, Chicago, ILL Company). Non-parametric distributed quantitative data are expressed as median and range. Categorical variables are expressed as numbers and percentages. Cumulative Incidence of EFS and OS were estimated using the Kaplan-Meier method. *P* value ≤ 0.05 was considered statistically significant.

## Results

### Patients’ characteristics: (Table 2)

Forty-one patients with newly diagnosed ALL were included in our study with a median age of the patients was 28 years (range 18–39 years). Male was the predominant gender as (68.29%) were males. Immunophenotyping study revealed that 30 patients (73.17%) were B-ALL. The cytogenetic study revealed 27 patients (65.85%) had normal karyotyping and eight patients (19.51%) were Philadelphia chromosome-positive. Allogeneic BMT from siblings was done for 10 patients (3 Philadelphia positive, 5 relapsed, and 2 with MRD). The remaining 5 Philadelphia chromosome-positive patients were not subjected to allogeneic BMT due to the lack of donors, these patients received tyrosine kinase inhibitors (imatinib or dasatinib).

**Table 2:** Characteristics of the study population

Variables	Median	Range
Age (years)	28	18–39
Hemoglobin (g/dl)	9	5–14
Total leucocytic count x10^9^/L	14	1–138
Platelets x10^9^/L	60	8–253
Bone marrow blast (%)	73	22–96
Variables		Number (%)
Sex	Male	28 (68.29%)
	Female	13 (31.71%)
Immunophenotyping	B-ALL	30 (73.17%)
	T-ALL	11 (26.83%)
Cytogenetics	Normal	27 (65.85%)
	Abnormal	14 (34.15%)
Philadelphia chromosome	Positive	8 (19.51%)
	Negative	33 (80.49%)

### Outcome results: (Table 3)

Thirty-eight patients (92.68%) achieved complete remission (CR), only 3 (7.32%) were refractory. Eleven patients (26.83%) relapsed (seven in the 1^st^ year, three in the 2^nd^ year, and one after that). Two patients out of the eleven relapsed patients had CNS relapse manifested by facial palsy and persistent headache with severe vomiting. Ten (24.39%) patients died (three just after induction after they became refractory and seven of them died after relapse).

**Table 3:** Therapy outcomes of the study population

Variables		Number (%)
Response	Complete remission (CR)	38 (92.68%)
	Refractory	3 (7.32%)
Relapse	Relapse	11 (26.83%)
	Non-relapse	30 (73.17%)
Survival	Living	31 (75.61%)
	Dead	10 (24.39%)

**Fig. 1:**
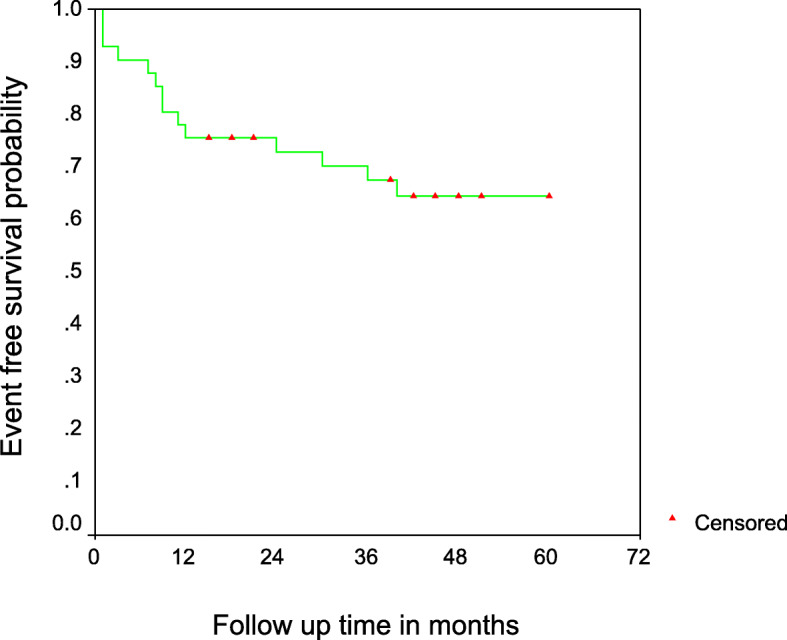
Kaplan–Meier analysis of the event free survival probability

**Fig. 2:**
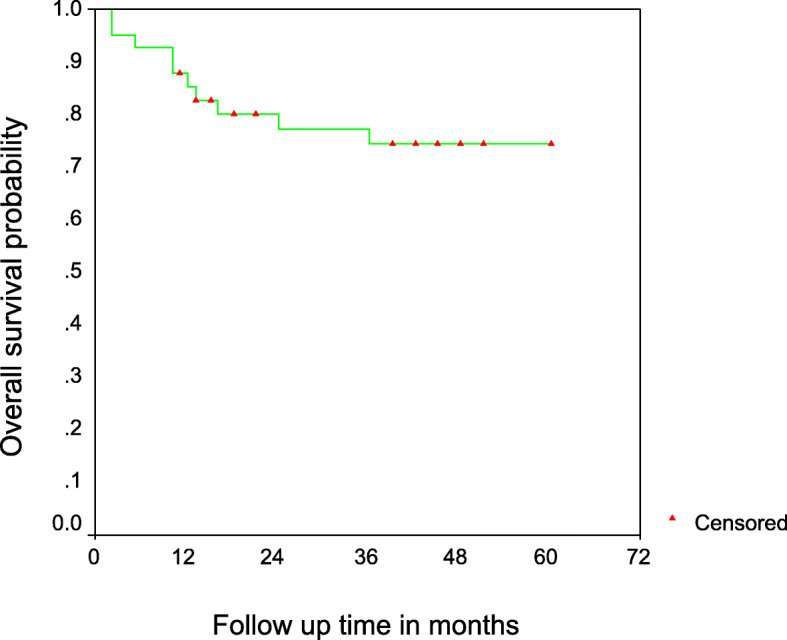
Kaplan–Meier analysis of the overall survival probability

The median follow-up period was 42 months (95% CI, 27.981–38.068). One-, two-, and three-year event-free survivals (EFS) were 75.61%, 72.91%, and 67.51% respectively (Fig. 1). One-, two-, and three-year overall survivals (OS) were 85.3%, 77.26%, and 74.39% respectively (Fig. 2).

### Toxicity: (Table 4)

All the patients (100%) had DFCP side effects; some patients had more than one side effect. No major side effects were observed that necessitated treatment discontinuation. Neutropenia was the most common adverse event observed in 41 patients (100%). Many other adverse events were also developed in patients under DFCP in the form of thrombocytopenia, febrile neutropenia, infections, hyperglycemia, mucositis, venous thromboembolism, neuropathy, hepatotoxicity, pancreatitis, and avascular necrosis. No other long-term side effects were developed during the follow-up period. No patient developed an allergy.

**Table 4:** Toxicity of Dana Farber Consortium Protocol in the study population

Toxicity	Number	Percentage (%)
Neutropenia	41	100
Thrombocytopenia	29	70.73
Febrile neutropenia	28	68.29
Infection	27	65.85
Hyperglycemia	10	24.39
Mucositis	9	21.95
Venous thromboembolism	4	9.76
Neuropathy	4	9.76
Hepatoxicity	4	9.76
Pancreatitis	3	7.31
Avascular necrosis	3	7.31

Raw data of all patients of the study were mentioned in (Table 5).

**Table 5:** Raw data of all patients of the study

No	Age ( years)	Sex	Total leucocytic count x10^9^/L	Hemoglobin (g/dl)	Platelets ×10^9^/L	Bone marrow blast (%)	Immunophenotyping	Philadelphia chromosome	Cytogenetics	Response	Relapsed	FATE
1	31	M	4	9	35	65	B	Negative	Normal	Refractory	N/A	Dead
2	23	M	13	13	223	66	B	Negative	Abnormal	Refractory	N/A	Dead
3	20	F	129	8.3	50	47	T	Negative	Abnormal	CR	No	Alive
4	31	M	77	10	55	96	B	Negative	Normal	CR	No	Alive
5	24	M	3	8.6	25	80	B	Negative	Normal	CR	No	Alive
6	32	M	14	12	18	72	B	Negative	Abnormal	CR	Yes	Dead
7	32	M	75	13	8	82	B	Positive	Abnormal	CR	Yes	Dead
8	21	M	97	7.6	53	70	B	Positive	Abnormal	CR	No	Alive
9	29	F	24	7.7	31	95	B	Negative	Normal	CR	No	Alive
10	26	M	7	13.6	206	42	T	Negative	Normal	CR	No	Alive
11	39	M	138	12.5	180	57	B	Negative	Normal	CR	Yes	Alive
12	27	F	4	9.2	27	95	T	Negative	Normal	CR	No	Alive
13	33	M	3	9	190	42	T	Negative	Normal	CR	Yes	Dead
14	18	M	10	11	98	25	T	Negative	Normal	CR	No	Alive
15	20	M	9	14	224	27	T	Negative	Normal	CR	No	Alive
16	28	F	4	9	160	80	T	Negative	Normal	CR	Yes	Alive
17	21	M	9	9	89	80	B	Positive	Abnormal	CR	No	Alive
18	26	M	3	5	80	91	B	Positive	Abnormal	CR	No	Alive
19	20	M	7	9.5	33	95	B	Negative	Normal	CR	No	Alive
20	27	M	41	8.7	50	94	B	Negative	Abnormal	CR	No	Alive
21	33	M	1	10	141	80	T	Negative	Normal	CR	Yes	Dead
22	22	M	6	7	196	83	T	Negative	Normal	CR	Yes	Alive
23	31	M	5	8	80	67	B	Negative	Normal	CR	Yes	Dead
24	23	M	17	8	53	90	T	Negative	Normal	CR	No	Alive
25	22	M	126	6.3	65	90	T	Negative	Normal	CR	No	Alive
26	35	M	5	6	60	85	B	Positive	Abnormal	CR	No	Alive
27	35	M	137	8.4	46	80	B	Negative	Normal	CR	No	Alive
28	34	F	88.2	8.3	57	90	B	Negative	Normal	CR	No	Alive
29	32	M	10.3	5.4	104	67	B	Negative	Normal	CR	No	Alive
30	30	F	90	7.2	60	87	B	Positive	Abnormal	CR	Yes	Dead
31	39	F	1	8.3	54	23	B	Negative	Abnormal	Refractory	N/A	Dead
32	33	F	43	9.6	13	73	B	Negative	Normal	CR	No	Alive
33	39	M	16	13.6	253	22	B	Negative	Normal	CR	Yes	Alive
34	23	M	138	8.7	46	90	B	Negative	Normal	CR	No	Alive
35	19	M	1.7	9.1	17	41	B	Negative	Normal	CR	No	Alive
36	30	F	52.9	8.5	40	90	B	Negative	Abnormal	CR	No	Alive
37	25	F	11.3	10.7	83	57	B	Negative	Normal	CR	No	Alive
38	31	F	38	8.9	55	37	B	Positive	Abnormal	CR	Yes	Dead
39	33	F	44	7.4	94	34	B	Positive	Abnormal	CR	No	Alive
40	27	F	63	10	121	61	B	Negative	Normal	CR	No	Alive
41	19	M	19	9.3	112	49	B	Negative	Normal	CR	No	Alive

*CR* complete remission, *F* female, *M* male, *N*/*A* not applicable

## Discussion

Adolescent and young adult (AYA) ALL patients represent a special patient group, as they may receive chemotherapy based on either pediatric or adult chemotherapy protocol [13]. The favorable outcomes found in children, coupled with unfavorable outcomes noticed in AYA, generated the idea for practical use of pediatric protocols among AYA. Also, many novel drugs such as Blinatumomab, inotuzomab, and chimeric antigen receptor T cell have been established to improve outcomes in poorly responding, relapsed, or refractory B cell ALL [14].

In ALL, AYA patients have poorer EFS and OS compared to children, because AYA tends to have unfavorable characteristics, such as T cell phenotype, more incidence of Philadelphia chromosome (9;22), and less occurrence of favorable chromosomal abnormalities such as hyperdiploidy [15]. In our study, immunophenotyping study revealed 26.83% of our patients were T-ALL, and 19.51% were Philadelphia chromosome-positive. These results coincide to a great extent with literatures, as in adults, T-ALL accounts for about 25% of cases, also Philadelphia chromosome in adults ALL can range from 15 to 50% increasing with age [16].

Several retrospective studies have shown that AYA patients who received pediatric protocols have better outcomes (CR 90–99% and EFS 63–80%) when compared with AYA patients who received adult protocols (CR 80–94% and EFS 34–71%) [17]. Although, one study by Usvasalo et al. [17] showed no superiority of both pediatric and adult protocols regarding CR and EFS.

Several factors may clarify better outcomes in pediatric protocols. Firstly, pediatric protocols have more non-myelosuppressive drugs with more activity on leukemic cells particularly during the BM suppression phase induced by anti-metabolites and anthracyclines. Moreover, in pediatric protocols, CNS prophylaxis as intrathecal methotrexate was administered earlier, more frequently, and for a longer time as CNS is the shelter for blast cells. Also, the maintenance therapy period is shorter in adult protocols [18, 19].

In our study, forty-one patients received the pediatric protocol DFCP, Thirty-eight patients (92.68%) achieved complete CR, eleven patients (26.83%) relapsed, and ten (24.39%) patients died. One-, two-, and three-year EFS were 75.61%, 72.91%, and 67.51% respectively. One-, two-, and three-year OS were 85.3%, 77.26%, and 74.39% respectively.

These results coincide to a great extent with some differences reported in many studies. DeAngelo et al. [20] used DFCP for the treatment of 74 adult patients (18–50 years) with ALL. Eighty-four percent of the patients achieved CR, with 2-year EFS and OS of 72.5% and 77.1%, respectively.

Barry et al. [11] treated 51 de novo ALL patients (15–18 years) with by DFCP, and found that the 5-year EFS and OS were 77.5% and 81% respectively. Also, Storring et al. [21] used a modified DFCP in 68 patients (17–71 years), 82% of the patients achieved CR, with 3-year EFS and OS of 77% and 65%, respectively.

Furthermore, Al-Khabori et al. [22] conducted a retrospective study on T-ALL patients, 32 AYA patients treated with a DFCP. Ninety-three percent of the patients achieved CR with 3-year relapse-free survival and OS 88% and 83% respectively. Besides, DeAngelo et al. [23] enrolled 92 patients (18–50 years) for a median follow-up period (4.5 years). Eighty-five percent achieved CR, with 4-year EFS and OS 69% and 67% respectively.

Lastly, Alabdulwahab et al. [24] enrolled 38 patients with a median age (19 years) or a median follow-up period (22 months). Then, 92.1% achieved CR, with 1- and 3-year EFS were 80% and 68%, respectively, and 1- and 3-year OS were 88% and 72%, respectively.

As ALL therapies have more intensified, more toxicities have been increased especially in AYA patients. The toxicities of intensified protocols increase in both incidence and severity due to hormonal changes, body weight changes, and different chemotherapy metabolism [25]. Researchers are focused on decreasing early and late toxicities by good use of supportive care to improve OS [26].

Pancreatitis and venous thromboembolism are usually linked with l-asparaginase use in DFCP but may be related to increased steroid dose and the use of dexamethasone [27, 28]. Avascular necrosis incidence was more experienced in DFCP trials using dexamethasone [29]. Hyperglycemia risk is more likely in the AYA patients of ALL due to adult hormonal changes, and is usually associated with immune suppression and the risk for infections [30].

In our study, all patients (100%) had developed side effects. Neutropenia was the most common adverse event observed in 100% of the patients. Many other adverse events were also developed in patients under DFCP in the form of thrombocytopenia (70.73%), febrile neutropenia (68.29%), infections (24.39%), hyperglycemia (24.39%), mucositis (21.95%), venous thromboembolism (9.76%), neuropathy (9.76%), hepatotoxicity (9.76%), pancreatitis (7.31%), and avascular necrosis (7.31%). No patient developed an allergy.

These results match with a great extent with some differences that were reported in many studies. DeAngelo et al. [20] shown in their study that the incidence of venous thromboembolism (19%) and pancreatitis (13%) but this drug-related toxicity was controllable.

DeAngelo et al. [23] demonstrated toxicities of DFCP included thrombocytopenia (82%), hepatic toxicity (62%), infection (61%), hyperglycemia (45%), febrile neutropenia (33%), thrombosis (17%), pancreatitis (11%), stomatitis (11%), bone fractures (8%), osteonecrosis (5%), allergy (5%), and CNS complications (5%).

Alabdulwahab et al. [24] demonstrated toxicities of DFCP included febrile neutropenia (100%), sepsis (29%), pneumonia (26%), typhlitis (21%), myopathy (13%), pancreatitis (13%), osteonecrosis (7.8%), neurological toxicity (5%), and severe liver failure together with renal failure (2.6%). There was no venous thromboembolism was recorded apart from (7%) who had central catheter-related thrombosis.

The discrepancy between results of our study and other previous studies could be explained by variations in number and age of patients, follow-up time, ALL phenotype (B or T), Philadelphia chromosome incidence, the occurrence of favorable, and unfavorable chromosomal abnormalities, ethnic differences, and regimens of chemotherapy used as DFCP has many modifications. To our knowledge, this is the first study to collect data from Egyptian ALL patients. The present study had some limitations such as a small number of patients and a short time of follow-up. Also, our study was retrospective; therefore, unrecognized biases might be considered. To overcome these limitations, further larger, longer, prospective, and multicenter studies are necessary.

## Conclusion

DFCP can be considered as an effective ALL protocol for the AYA group of patients with a good complete remission, event-free survival, and overall survival rates. DFCP seemed to be feasible in AYA despite the toxicities experienced which overcame by good supportive care and temporary cessation of some drugs.

## Data Availability

All data generated or analyzed during this study are included in this published article.

## Abbreviations

ALL: Acute lymphoblastic leukemia
AYA: Adolescents and young adults
BCR-ABL1: Fusion between BCR gene (Breakpoint Cluster Region) and ABL1 gene (ABelson murine Leukemia virus)
BM: Bone marrow
BMT: Bone marrow transplantation
CBC: Complete blood count
CNS: Central nervous system
CR: Complete remission
DFCP: Dana Farber Consortium Protocol
ECOG: Eastern Co-operative Oncology Group
EFS: Event-free survival
MRD: Minimal residual disease
OS: Overall survival
SPSS: Statistical Package for the Social Sciences
t: Translocation
